# Numerical optimization of TiO_2_/SnO_2_ bilayer electron transport layers for enhanced perovskite solar cell performance

**DOI:** 10.1186/s11671-025-04357-w

**Published:** 2025-09-15

**Authors:** Haoran Ma, Yajun Xu, Jun Zhao, Jun Wu, Luanhong Sun, Jinjie Zheng, Wei Zhang

**Affiliations:** 1 School of Electrical and Energy Engineering, Key Laboratory of Optoelectronic Materials, Nantong Institute of Technology, Nantong, 226002 Jiangsu People’s Republic of China; 2 College of Physics, Nanjing University of Aeronautics and Astronautics, Nanjing, 211106 People’s Republic of China; 3 Suzhou Huaqinyuan Microelectronics Technology Co. Ltd, Suzhou, 215600 Jiangsu People’s Republic of China; 4 School of Materials Engineering, Jinling Institute of Technology, Nanjing, 211169 People’s Republic of China

## Abstract

To improve charge extraction and address UV-induced degradation in perovskite solar cells, we propose and numerically evaluate a TiO_2_/SnO_2_ bilayer electron transport layer (ETL) architecture. Using physics-based simulation, we systematically analyze the influence of individual and combined ETL thicknesses on key parameters. The results identify an optimal configuration of 100 nm TiO_2_ and 20 nm SnO_2_, which minimizes interfacial recombination and enhances electron transport. Furthermore, CH_3_NH_3_SnI_3_ is employed as a lead-free absorber layer. Simulation results demonstrate a notable efficiency improvement upto 20.80%. The experimental results verified that the bi-layer Sn-based perovskite can achieve a conversion efficiency of 10.3%. This study highlights the potential of simulation-guided design in optimizing multilayer ETL structures and advancing environmentally friendly, high-efficiency perovskite photovoltaics.

**Supplementary Information:**

The online version contains supplementary material available at 10.1186/s11671-025-04357-w.

## Introduction

As a hallmark of third-generation photovoltaic technology, Single-junction perovskite solar cells have emerged as a pivotal focus in renewable energy research, driven by their exceptional optoelectronic properties (certified Eff up to 27.3%) [1], cost-effective solution processability, and tunable bandgap engineering. Within this framework, the light-absorbing layer serves as the cornerstone of device performance, where material innovation dictates efficiency breakthroughs. While conventional lead-based (Pb^2+^) perovskites exhibit superior photoconversion capabilities, their practical deployment is impeded by inherent lead toxicity and environmental persistence.

Recent advancements demonstrate that main-group metal ions (e.g. Ge^2+^, Sn^2+^) can effectively substitute Pb^2+^ to construct eco-compatible perovskite architectures [2]. Tin-based variants, in particular, stand out as the most viable alternative, owing to their narrow bandgap (1.2–1.4 eV) [3] and exceptional charge carrier mobility (> 200 cm^2^ V^− 1^ s^− 1^) [4]. Noel et al. pioneered 6%-efficient tin-based devices, this field has evolved through multidimensional optimizations [5]. Liu et al. enhanced efficiency to 9.06% via interfacial engineering [6]. Xin et al. achieved 9.47% through solvent engineering [7]. Significantly, certified efficiencies for state-of-the-art tin-based perovskites optimized for defect passivation and crystalline control now exceed 14%, which represents a significant milestone in competing with their lead-based counterparts [8]. In addition, the complexity of Sn-based perovskite devices urgently requires advanced modelling techniques to reveal their underlying physical mechanisms and optimise their performance, as exemplified by the comprehensive simulation study conducted by Kumar Neupane et al.

The perovskite electron layer, which acts as a medium for light contact with the cell, is an important indicator of the Efficiency of perovskite cells. The degree of light absorption by the perovskite electron layer can directly affect the performance of the perovskite cell. For instance, recent SCAPS-1D simulations on Rb-based halide perovskite solar cells demonstrated that different ETL materials (e.g., TiO_2_, SnO_2_, WS_2_) lead to substantial variations in PCE, highlighting the crucial role of ETL selection [9]. The common single-electron layer material TiO_2_ is photocatalytically degraded under ultraviolet light, and the environmental conditions for crystallisation are more stringent, making it difficult to apply TiO_2_ in flexible and stacked cells. For example, some researchers have replaced the defect that TiO_2_ is photocatalytically degraded under UV light by using Fe_2_O_3_, an electronic layer with low photocatalytic activity, but the narrow bandgap of Fe_2_O_3_ makes the cell less capable of electron extraction [10]. The emergence of double electron layers can solve some problems that many materials cannot avoid as single electron layers. Li et al. investigated a double electron layer cell with a TiO_2_/SnO_2_ combination prepared by a low-temperature solution method. This combination reduces the barrier height by adjusting the structure of the energy bands, thus enhancing the electron transport ability [11]. M. I. Khan et al. increased the crystal size and improved the crystallinity by nickel-doped WO_3_ combined with TiO_2_ to form a double electron layer to enhance the electron transport performance of the electron layer, which improved the light absorption Efficiency and the J-V Efficiency was also greatly improved [12]. Liu et al. designed a Mn quantum dot sandwiched in the middle of the double electron layer to reduce the interfacial energy level shift, improve electron transport, and increase the Efficienc of the cell to 24.63% [13].

Dual ETL architectures have also been explored in lead-free perovskite systems, where combining two ETLs (e.g., MXene + TiO_2_) significantly enhanced PCE and FF by improving interfacial properties and reducing recombination [14]. Therefore, we combine two perovskite electron layer materials, TiO_2_ and SnO_2_, as a double electron layer, and deeply investigate the four electrical performance indexes of the two materials, namely, short-circuit current density (J_sc_), open-circuit voltage (V_oc_), fill factor (FF), and Eff, in the interval of 20–100 nm with the thickness. The thickness variation trend of the four electrical performance indexes in the interval of 20–100 nm with the thickness change, screening out the optimal thickness of the two materials in the interval and combining them to obtain the optimal thickness of the double electron layer combination. For the lead-free perovskite absorber layer, CH_3_NH_3_SnI_3_ was chosen to carry out the study, and its cell performance was compared with that of CH_3_NH_3_PbI_3_ as the perovskite absorber layer.

## Experimental section

### Materials and simulation

The semiconductor carrier equations applied for the simulation are shown in Eqs. (1)–(4):

1 $$\:\nabla\:\cdot\:{\text{J}}_{\text{n}}\text{}=-\text{q}{\text{R}}_{\text{n}}$$

2 $$\:\nabla\:\cdot\:{\text{J}}_{\text{p}}=\text{q}{\text{R}}_{\text{p}}$$

where ∇·J_n_ and ∇⋅J_p_ denote the scattering of the current densities of electrons and holes, respectively, q is the electron charge, and R_n_ and R_p_ are the complexation rates of electrons and holes, respectively.

3 $$\:{\text{R}}_{\text{n}}=\text{C}\left(\text{n}\text{p}-{{\upgamma\:}}_{\text{n}}{{\upgamma\:}}_{\text{p}}{\text{n}}_{\text{i},\text{e}\text{f}\text{f}}^{2}\right)={\text{R}}_{\text{p}}$$

where R_n_ and R_p_ are the electron and hole complexity, which are usually assumed to be equal, and C is the complexity coefficient. n and p are the concentrations of electrons and holes, respectively. γ_n_ and γ_p_ are the simplex factors of electrons and holes. n_i, eff_ are the effective intrinsic carrier concentrations.

4 $$\:{\text{n}}_{\text{i},\text{e}\text{f}\text{f}}=\sqrt{{\text{N}}_{\text{c}0}{\text{N}}_{\text{v}0}}\text{e}\text{x}\text{p}(-\frac{{\text{E}}_{\text{g}}-\varDelta\:{\text{E}}_{\text{g}}}{2{\text{K}}_{\text{B}}\text{T}})$$

N_c0_ and N_ν0_ are the effective density of states in the conduction and valence bands, respectively, E_g_ is the bandgap energy of the semiconductor, ΔE_g_ is the change in bandgap energy (e.g., due to strain or other effects), K_B_ is the Boltzmann constant, and TT is the absolute temperature.

**Fig. 1:**
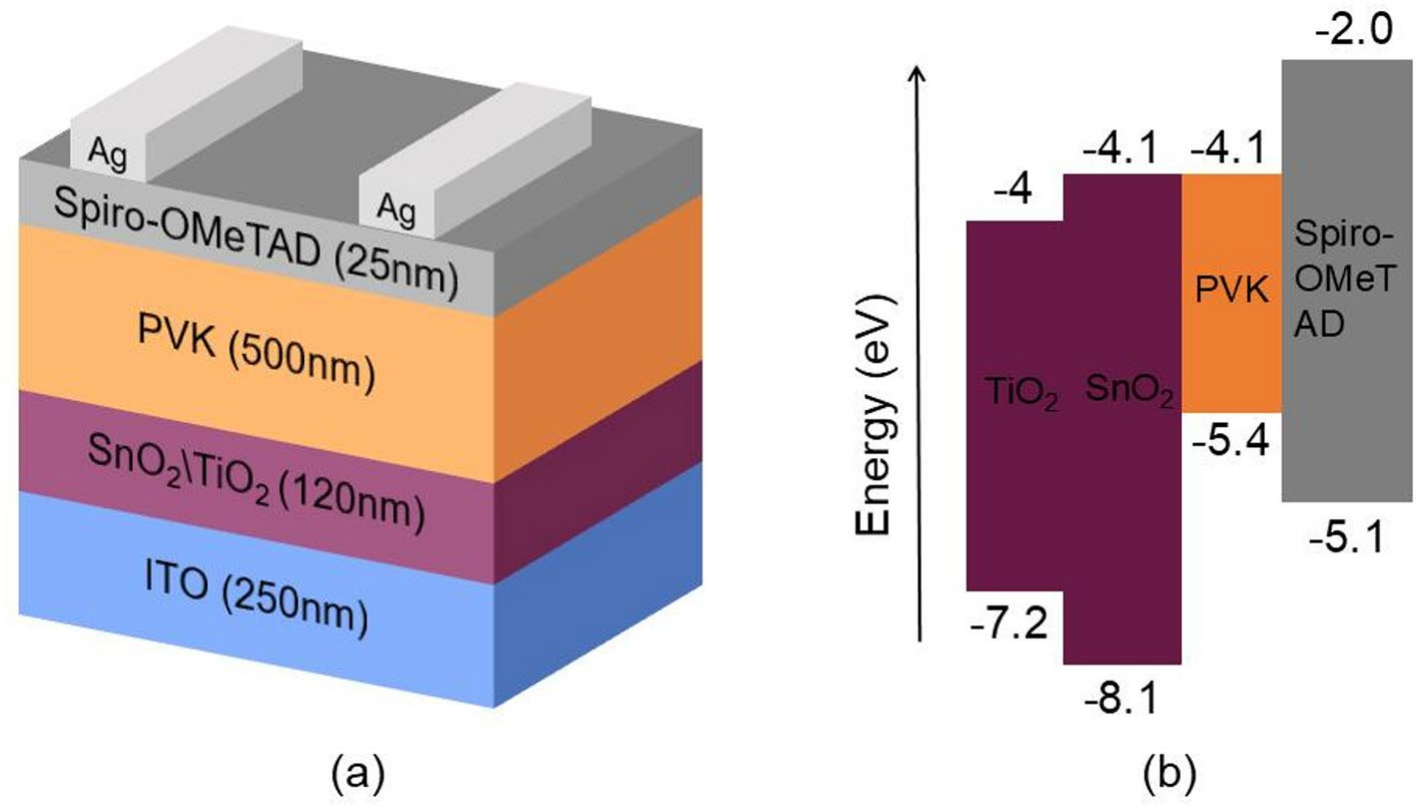
**a** The cell structure and **b** Material band diagram

The general perovskite cell consists of electrode, ETL, hole transport layer (HTL), perovskite absorber layer, and conductive substrate. In this paper, Ag is chosen as the electrode, the combination of TiO_2_ and SnO_2_ as the ETL, Spiro-OMeTAD as the hole transport layer, CH_3_NH_3_SnI_3_ as the perovskite absorber layer, and ITO as the conductive substrate. ITO is used as the conductive substrate. Figure 1 shows the cell structure and energy level structure. The calculated parameters of each material layer are shown in Table 1.

**Table 1:** Input parameters for ETL and CH_3_NH_3_SnI_3_ absorber layer

Parameter	TiO_2_ [15, 16]	SnO_2_[17]	CH_3_NH_3_SnI_3_ [18, 19]	Spiro-OMeTAD [18, 20]
Layer thickness/nm	100	20	500	25
Bandgap/eV	3.2	3.5	1.30	3
Electron affinity	4	4	4.17	2.2
Relative permittivity	9	9	8.2	3
Effective conduction band density/cm^−3^	2.2e18	3.7e18	2e18	2e18
Effective valence band density/cm^−3^	1e19	1.8e19	1e19	1.8e19
Electron mobility /(cm^2^/V^−1^/S^−1^)	20	20	1e7	100
Hole mobility /(cm^2^/V^−1^/S^−1^)	10	10	1e7	2

### Synthesis of ch_3_nh_3_sni_3_ thin films

The CH_3_NH_3_SnI_3_ perovskite absorber layer was synthesized via a one-step spin-coating method. The precursor solution was prepared by dissolving 1 M methylammonium iodide (MAI) and 1 M tin(II) iodide (SnI_2_) in a mixed solvent of dimethylformamide (DMF) and dimethyl sulfoxide (DMSO) at a volume ratio of 4:1. To improve film crystallinity and reduce Sn(IV)-related oxidation, a small amount of SnF_2_ (10 mol%) was added as an additive. The solution was stirred at 60 °C for 2 h in a nitrogen-filled glovebox to ensure full dissolution and minimize oxygen exposure. The resulting solution was filtered and spin-coated onto the ETL at 4000 rpm for 30 s. During spin coating, an anti-solvent (chlorobenzene) was dropped 10 s after spin initiation to induce rapid crystallization. The wet film was immediately annealed at 100 °C for 10 min to complete perovskite film formation. All processing steps were conducted under inert atmosphere to preserve the stability of the Sn-based perovskite layer [5, 21]. The CH_3_NH_3_SnI_3_ thin film synthesis method described in this section provides detailed guidance on thin film preparation for this work. Since the focus of this study is on simulation, only the synthesis description is included to provide background information on the material parameters used in the simulation. Complete double-layer devices were not prepared in this study, only a single-layer reference was made.

## Result and discussion

### The study of the single ETL layer

**Fig. 2:**
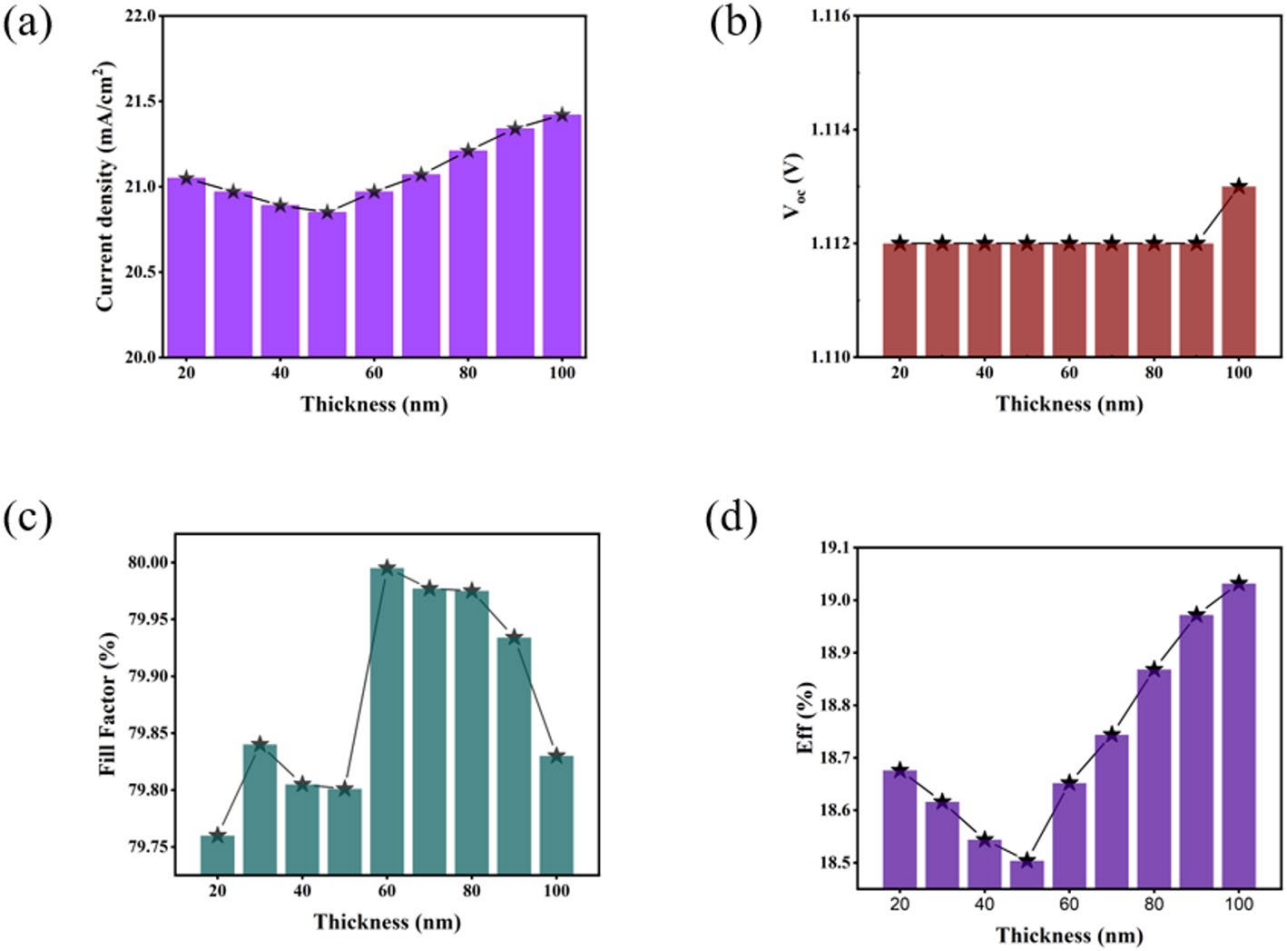
Variation curves of perovskite solar cells **a** J_sc_, **b** V_oc_, **c** FF, **d** Eff with the thickness of TiO_2_ electron layer

TiO_2_ is a widely used ETL in perovskite solar cells. Using the structure and parameters in Table 1, we simulated performance versus TiO_2_ thickness (20–100 nm). At 20–50 nm, incomplete coverage can expose the perovskite to the conductive substrate, enhancing interfacial recombination and degrading charge extraction. Beyond ~ 50 nm, improved film continuity suppresses interfacial recombination, and the favorable band alignment aids electron extraction, leading to gradual improvements in J_sc_ and efficiency. The observed J_sc_ exhibits a non-monotonic dependence on TiO_2_ thickness, decreasing from 20 to 50 nm before increasing between 50 and 100 nm. This phenomenon can be attributed to interfacial discontinuity in ultra-thin films (20–50 nm), where incomplete coverage of the photoactive layer permits direct contact between the perovskite absorber and conductive substrate, inducing severe interfacial recombination and impairing carrier extraction Efficiency. Furthermore, marginal thickness increases within this range extend electron transport pathways while accentuating the detrimental effects of intrinsic resistance components, particularly grain boundary resistance, thereby exacerbating charge transport losses. As the TiO_2_ thickness exceeds 50 nm, improved film continuity and densification effectively isolate the perovskite layer from the conductive substrate, suppressing interfacial recombination [22]. Concurrently, the favorable band alignment of TiO_2_ enhances electron extraction capability, thereby improving carrier separation Efficiency. V_oc_ remains relatively stable with only a 1 mV increase at 100 nm thickness, consistent with simulation results indicating that V_oc_ primarily correlates with material bandgap - an intrinsic property minimally affected by dimensional variations in the ETL. Fill Factor shows a complex thickness dependence, initially increasing between 20 and 30 nm due to a decrease in parallel resistance, which improves the charge transport Efficiency, and then gradually decreases as the intrinsically low conductivity of TiO_2_ restricts the migration of electrons through the extended transport path. A maximum FF occurs at 60 nm thickness, corresponding to an optimal balance between charge transport efficiency and optical absorption characteristics. Beyond 70 nm, excessive layer thickness promotes intensified carrier recombination, leading to sustained FF reduction [23–25]. Notably, power conversion efficiency demonstrates continuous improvement from 60 nm to 100 nm, potentially attributable to the optical spacer effect of thicker TiO_2_ layers. This phenomenon enhances light field distribution through interference effects, particularly improving long-wavelength photon absorption in the perovskite layer, thereby compensating for transport-related losses and ultimately increasing J_sc_ (Fig. 2).

**Fig. 3:**
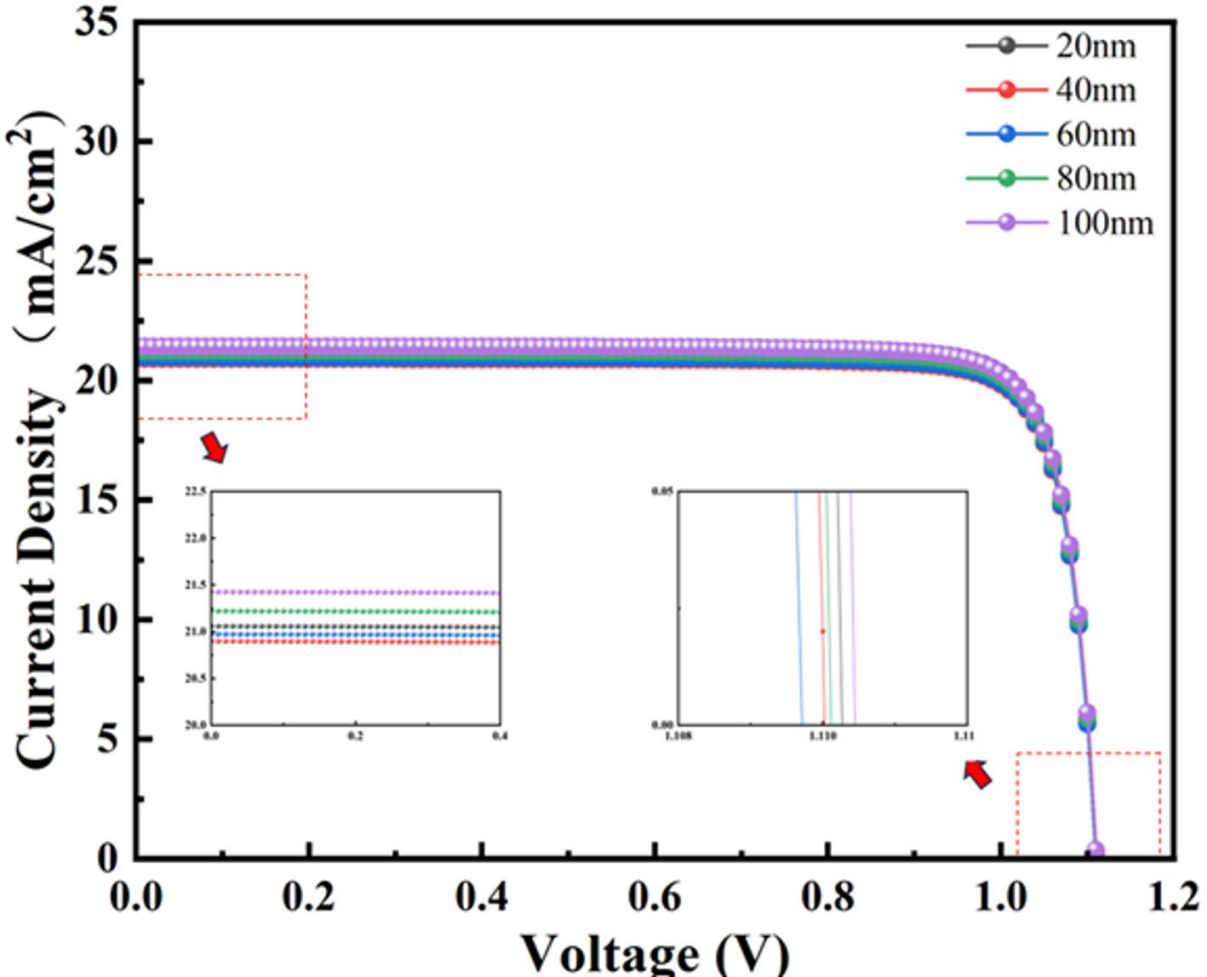
J-V curves of titanium dioxide as PVK solar cells at PVK layer thicknesses of 20–100 nm (the rest are shown in Fig. S2)

A systematic investigation into the thickness-dependent characteristics of ETL was conducted to elucidate its impact on photovoltaic performance, as demonstrated in Fig. 3. Analysis reveals that the device achieves maximum J_sc_ at 100 nm ETL thickness, coinciding with its peak optical absorption Efficiency. This correlation is attributed to enhanced light-harvesting capabilities and optimized charge extraction dynamics at greater ETL dimensions. The experimental data confirm a monotonic improvement in power conversion Efficiency across the 20–100 nm thickness range for TiO_2_-based single ETL configurations, as shown in Fig. S1. This trend corroborates the critical role of ETL thickness in balancing interfacial recombination suppression and charge transport optimization, where progressive thickness increases facilitate both improved perovskite/ETL interface passivation and favorable energy band alignment for carrier collection. Notably, the continuous Efficiency enhancement despite FF reduction beyond 60 nm thickness underscores the dominance of optical management over transport losses in thicker ETL architectures, particularly through enhanced light trapping and spectral utilization efficiency.

**Fig. 4:**
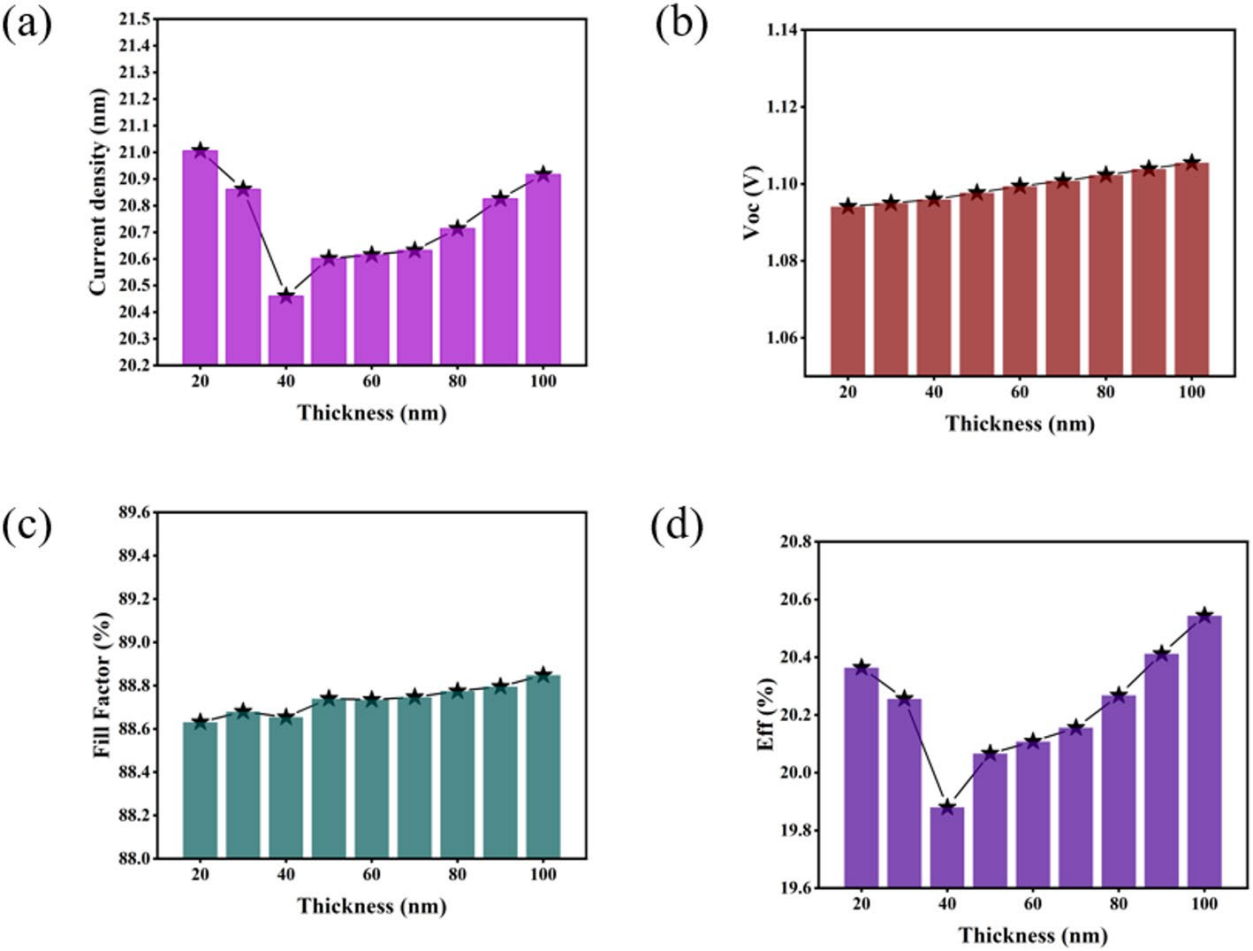
Variation curves of perovskite solar cells **a** J_sc_, **b** V_oc_, **c** FF, **d** Eff with the thickness of SnO_2_ electron layer

Recent investigations have highlighted SnO_2_ as a promising ETL material for perovskite solar cells owing to its exceptional electron mobility, wide bandgap, and superior energy level alignment [26]. In this study, SnO_2_ was systematically evaluated as a single ETL material through comprehensive simulation analysis(see Fig. S3 for absorption rates). Figure 4 illustrates the thickness-dependent evolution of four critical electrical parameters across the same dimensional range previously analyzed for TiO_2_ (20–100 nm), revealing distinct performance characteristics. Unlike the TiO_2_-based system, SnO_2_ demonstrates a pronounced thickness-dependent enhancement in V_oc_, exhibiting a continuous increase as ETL thickness grows. This improvement originates from the progressive formation of a fully continuous SnO_2_ film that effectively isolates the perovskite layer from the conductive substrate at sufficient thickness (> 60 nm), thereby suppressing interfacial recombination and optimizing energy band alignment. The combined effects of enhanced V_oc_ and notably improved Fill Factor establish SnO_2_ as a technologically advantageous ETL material, demonstrating thickness-dependent performance optimization mechanisms distinct from conventional TiO_2_-based architectures.

### The study of the TiO_2_/SnO_2_ bilayer

While SnO_2_ has emerged as an established ETL material in perovskite photovoltaics, conventional TiO_2_-based systems exhibit suboptimal charge transport characteristics due to the inherent disparity between hole diffusion length and electron transport capacity, creating charge extraction imbalance [27]. This performance limitation is further exacerbated by substantial interface defect states at the TiO_2_/perovskite boundary, which promote deleterious carrier recombination and associated energy losses [28, 29]. Recent advancements in interfacial engineering strategies demonstrate that these limitations can be effectively mitigated through innovative dual-ETL architectures. The synergistic combination of tin oxide and titanium oxide in the bilayer configuration exploits the superior electron extraction Efficiency of tin oxide and the proven stability of titanium oxide to form a cascade energy arrangement that enhances charge transport kinetics while inhibiting interfacial recombination. Table 2 provides a quantitative comparison of key photovoltaic parameters between single-layer and dual-ETL configurations, revealing that the bilayer structure achieves remarkable improvements in both V_oc_ (through interface defect passivation) and Fill Factor. This architectural optimization addresses the fundamental limitations of single-ETL systems by decoupling interface engineering requirements from bulk transport optimization, enabling independent control over recombination suppression and carrier collection efficiency [30]. 

**Table 2:** PV parameters of 20 nm SnO_2_ and 100 nm TiO_2_

Parameter	J_sc_ (mA/cm^2^)	V_oc_ (V)	FF (%)	Eff (%)
SnO_2_	21.27	1.057	88.13	20.27
TiO_2_	21.42	1.098	79.83	19.03

The comparative analyses in Table 2 reveal distinct electrical behaviors for perovskite solar cells incorporating TiO_2_ and SnO_2_ as single-layer ETLs under otherwise identical simulation conditions. The TiO_2_-based device exhibits a marginally higher open-circuit voltage (1.098 V) and short-circuit current density (21.42 mA/cm^2^), likely due to its favorable energy band alignment and stable interfacial properties. However, SnO_2_ demonstrates a significantly higher fill factor of 88.13% compared to 79.83% for TiO_2_, indicating more efficient charge extraction and reduced recombination losses. As a result, despite slightly lower Voc and Jsc, the SnO_2_-based cell achieves a notably higher efficiency of 20.27%, surpassing the 19.03% of its TiO_2_ counterpart. These results validate the potential of SnO_2_ as a superior ETL material in terms of overall device performance, especially when fill factor dominates efficiency contribution. The findings also corroborate the earlier thickness-dependent optimization, highlighting SnO_2_’s superior compatibility with the perovskite interface and its ability to maintain excellent transport characteristics even at thinner configurations. The conclusion that can be drawn is that the efficiency of the optimised two-electron layer cell is improved compared to the cell efficiency of the two monolayer materials, which confirms the feasibility of the simulation of the two-electron layer cladding cell combining the two materials in this study.

Although the simulated PCE improvement from 20.276% (SnO_2_ ETL) to 20.800% (TiO_2_/SnO_2_ bilayer ETL) is modest, the bilayer structure provides meaningful physical advantages beyond the absolute efficiency value. In our analysis, introducing the TiO_2_ interlayer reduces the interfacial trap density from the order of 1 × 10^16^ cm^− 3^ toward ~ 1 × 10^15^ cm^− 3^, which lies within the typical range reported for high-quality perovskite films [31]. The lower trap density suppresses Shockley-Read-Hall (SRH) recombination at the perovskite/ETL interface and thereby benefits V_oc_. In addition, the cascade energy-level alignment between TiO_2_ and SnO_2_ facilitates more efficient electron extraction and alleviates charge accumulation; accordingly, we modeled a reduction of the effective series resistance from ~ 5–8 Ω·cm^2^ toward ~ 3 Ω·cm^2^ to reflect improved interfacial transport. While these changes do not produce a dramatic rise in simulated PCE, they are expected to enhance device stability, mitigate hysteresis, and improve long-term reproducibility, consistent with recent reports on TiO_2_/SnO_2_ bilayer ETLs [32].

**Fig. 5:**
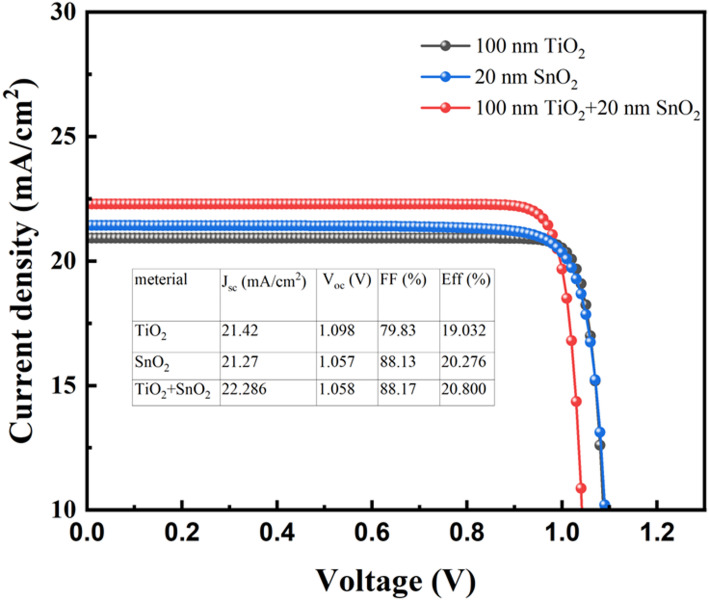
J-V curves for a bilayer ETL device and a single electron layer of TiO_2_ and SnO_2_

### The study of TiO_2_/SnO_2_ bilayer on Sn-based perovskite

The PVK layer plays a pivotal role in determining photovoltaic performance through its optoelectronic characteristics and environmental compatibility. While lead-based PVK materials dominate conventional solar cell architectures, their intrinsic toxicity and environmental persistence raise substantial concerns regarding sustainable implementation. We used CH_3_NH_3_SnI_3_ as a alternative, taking advantage of the comparable ionic radii of tin and similar coordination chemistry with halides to address toxicity concerns while maintaining good bandgap properties [33]. It has been observed that Sn has better light absorption in the visible region as well as a similar ionic radius to Pb, which means that Sn has similar chemical properties to Pb. With similar chemical properties, CH_3_NH_3_SnI_3_ is chosen as the Sn-containing perovskite absorber layer layer in this simulation, and the difference in the optical performance of the cell between this material as a perovskite absorber layer and the conventional lead-containing perovskite absorber layer is also investigated in the simulation in this paper, as shown in Fig. 5; Table 3 shows the electrical parameters of these two different PVK layers.

Table 3 presents the simulated photovoltaic parameters of perovskite solar cells utilizing CH_3_NH_3_SnI_3_ and CH_3_NH_3_PbI_3_ as light absorbers in combination with a TiO_2_ (100 nm)/SnO_2_ (20 nm) bilayer ETL. The Sn-based device achieve J_sc_ of 22.286 mA/cm^2^, V_oc_ of 1.058 V, and FF of 88.17%, resulting in Eff of 20.80%. For the Pb-based counterpart, the FF is significantly lower at 84.00%, likely due to increased recombination or resistance losses under idealized simulation assumptions. However, the Pb-based cell compensates with a higher V_oc_ of 1.135 V and a slightly lower J_sc_ of 21.900 mA/cm^2^, ultimately achieving a marginally improved Eff of 20.88%. These results reflect the intrinsic trade-offs between voltage, current density, and FF in determining overall device performance and highlight the importance of interface and absorber energy alignment.

**Table 3:** Simulated electrical parameters of two different PVK layers

Device	J_sc_(mA/cm^2^)	V_oc_ (V)	FF (%)	Eff (%)
CH_3_NH_3_SnI_3_	22.286	1.058	88.17	20.80
CH_3_NH_3_PbI_3_	21.900	1.135	84.00	20.88

**Fig. 6:**
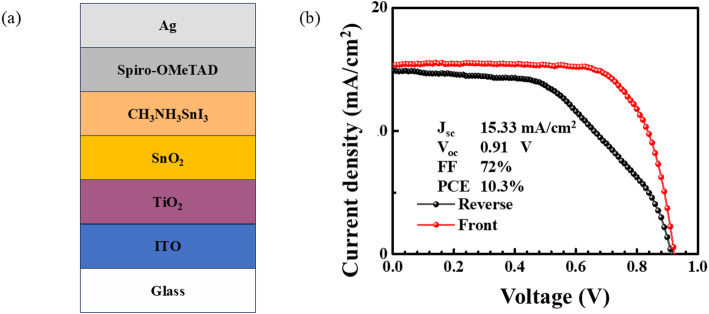
**a** Chematic of the experimental CH_3_NH_3_SnI_3_ device. **b** Forward and reverse J–V curves

The results in Table 3 indicate that the simulated performance of the CH_3_NH_3_SnI_3_-based device reaches Eff of 20.80% (The structure of the cell is shown in Fig. 6(a).), with high fill factor and current density under ideal conditions. In comparison, the experimentally fabricated device exhibits a significantly lower efficiency, as evidenced by the J–V curve shown in Fig. 6(b). Despite the gap between simulated and experimental values, the overall trends in open-circuit voltage and fill factor are directionally consistent, confirming the validity of the model assumptions; however, the significant efficiency discrepancy warrants a more detailed analysis of the performance loss mechanisms.

Although CH_3_NH_3_SnI_3_-based devices with a TiO_2_/SnO_2_ double-layer ETL achieved a photoconversion efficiency of 20.80% in simulations, experimentally fabricated devices only reached an efficiency of 10.3%. This significant discrepancy is primarily due to various non-ideal factors in the actual fabrication process. The ideal simulated environment did not adequately account for recombination losses caused by interface and bulk defects. The tin-based perovskite layer and its interfaces in the experiments exhibited high trap densities, significantly enhancing Shockley-Read-Hall(SRH) recombination, leading to reduced open-circuit voltage and fill factor. Additionally, Sn^2+^ readily oxidises to Sn^4+^, even when processed in an inert atmosphere, introducing deep-level defects and p-type doping, further reducing carrier lifetime and short-circuit current density. Significant series resistance is also present in the experimental devices, originating from non-ideal ohmic contacts and interface barriers, severely affecting charge extraction efficiency. Additionally, spin-coated perovskite films may exhibit incomplete coverage, pinholes, or rough morphology, leading to current leakage and loss of effective active area. The simulations also did not account for parasitic absorption by the ETL or electrodes, nor the photon loss due to insufficient optical coupling. Furthermore, the environmental sensitivity of tin-based perovskites, which degrade rapidly during measurement, collectively result in experimental performance far below theoretical values. Future efforts should focus on addressing these gaps through interface passivation, deposition process optimisation, antioxidant addition, and enhanced encapsulation strategies.

## Conclusion

In this work, a numerical simulation framework was developed to systematically investigate the thickness-dependent performance of ETLs in perovskite solar cells with varied absorber compositions. The simulations reveal that TiO_2_ at a thickness of 100 nm and SnO_2_ at a thickness of 20 nm achieve optimal performance in single-layer configurations, with respective efficiencies of 19.03% and 20.27%. By integrating the two materials into a bilayer ETL structure, an enhanced efficiency of 20.80% was obtained under ideal simulation conditions, attributed to improved energy band alignment and reduced interfacial recombination. Furthermore, the comparison between CH_3_NH_3_SnI_3_ and conventional lead-based CH_3_NH_3_PbI_3_ absorbers highlighted the trade-off between different absorber materials, highlighting the trade-off between higher fill factor in Sn-based devices and higher efficiency in Pb-based devices. While experimental results confirmed key trends predicted by the simulations, discrepancies due to interfacial defects emphasize the need for further optimization in practical device fabrication. This study provides quantitative guidance for ETL structure design and supports the development of environmentally friendly, high-performance perovskite solar cells.

## Supplementary Information

Below is the link to the electronic supplementary material.

Supplementary Material 1

## Data Availability

The datasets generated and analyzed during the current study are available from the corresponding author on reasonable request. Simulation data were obtained using custom models and parameters described in the manuscript, and experimental measurements are available in the figures and tables provided.

## Declarations

### Clinical trials registration

This study does not involve any human or animal subjects and is not applicable to clinical trial registration.

## References

[1] Green MA et al. Solar cell efficiency tables (Version 66). Prog Photovolt. 2025;33:795–810. 10.1002/pip.3919.

[2] Wu T, et al. Lead-free Tin perovskite solar cells. Joule. 2021;5:863–86.

[3] Stoumpos CC, Malliakas CD, Kanatzidis MH. MG Semiconducting Tin and lead iodide perovskites with organic cations: phase transitions, high mobilities, and near-infrared photoluminescent properties. Inorg Chem. 2013;52:9019–38. 23834108 10.1021/ic401215x

[4] Gu S, et al. Tin and mixed lead–tin halide perovskite solar cells: progress and their application in tandem solar cells. Adv Mater. 2020;32:1907392. 10.1002/adma.201907392 32053273

[5] Noel NK, et al. Lead-free organic–inorganic Tin halide perovskites for photovoltaic applications. Environ Sci. 2014;7:3061–8.

[6] Liu X, et al. Solvent engineering improves efficiency of lead-free tin-based hybrid perovskite solar cells beyond 9%. ACS Energy Lett. 2018;3:2701–7.

[7] Yu BB, et al. Heterogeneous 2D/3D tin-halides perovskite solar cells with certified conversion efficiency breaking 14%. Adv Mater. 2021;33:2102055. 10.1002/adma.202102055 34296476

[8] He X, et al. Highly efficient Tin perovskite solar cells achieved in a wide oxygen concentration range. J Mater Chem A. 2020;8:2760–8.

[9] Neupane K et al. Empowering rubidium-based halide pscs: a deep dive into ETL material performance. J Phys Chem Solids 2025;112897.

[10] Zuo L, et al. Enhanced photovoltaic performance of CH_3_NH_3_PbI_3_ perovskite solar cells through interfacial engineering using self-assembling monolayer. J Am Chem Soc. 2015;137:2674–9. 25650811 10.1021/ja512518r

[11] Li N, et al. A low-temperature TiO_2_/SnO_2_ electron transport layer for high-performance planar perovskite solar cells. Sci China Mater. 2020;63:207–15.

[12] Khan M, et al. Enhancing the structural and optoelectronic properties of double ETL nickel-doped CsPbIBr_2_ perovskite solar cells. CrystEngComm. 2024;26:3535–46.

[13] Liu B, et al. Perovskite solar cells with extremely high 24.63% efficiency through design of double electron transport layers and double luminescent converter layers. Adv Funct Mater. 2024;34:2401007.

[14] Neupane K, et al. Exclusive optimization of Cs2AgBi0. 75Sb0. 25Br6-based solar cells using dual ETL with better photo transmission. Multiscale Multidiscip Model Exp Des. 2025;8:1–13.

[15] Rai S, Pandey B, Dwivedi D. Device simulation of low cost HTM free perovskite solar cell based on TiO_2_ electron transport layer. AIP Conference Proceedings 2220 (2020).

[16] Kumar M, Raj A, Kumar A, Anshul A. Computational analysis of bandgap tuning, admittance and impedance spectroscopy measurements in lead-free MASnI_3_ perovskite solar cell device. Int J Energy Res. 2022;46:11456–69.

[17] Li Q, Gan Y, Tan B, Mo P, Jiang Q. Numerical simulation of perovskite solar cells based on Cu_2_O and SnO_2_. Chin J Power Sources. 2020;44:1321–3.

[18] Imani S, Seyed-Talebi SM, Beheshtian J, Diau EWG. Simulation and characterization of CH_3_NH_3_SnI_3_-based perovskite solar cells with different Cu-based hole transporting layers. Appl Phys A. 2023;129:143.

[19] Srivastava S, Singh AK, Kumar P, Pradhan BJ. Comparative performance analysis O. lead-free perovskites solar cells by numerical simulation. J Appl Phys 131 (2022).

[20] Jayan KD, Sebastian VJSE. Comprehensive device modelling and performance analysis of MASnI_3_ based perovskite solar cells with diverse ETM, HTM and back metal contacts. Sol Energy. 2021;217:40–8.

[21] Hao F, Stoumpos CC, Cao DH, Chang RP, Kanatzidis MG. Lead-free solid-state organic–inorganic halide perovskite solar cells. Nat Photonics. 2014;8:489–94.

[22] Huang H, et al. 24.8%-efficient planar perovskite solar cells via ligand-engineered TiO2 deposition. Joule. 2022;6:2186–202.

[23] Kim H-S, et al. Lead iodide perovskite sensitized all-solid-state submicron thin film mesoscopic solar cell with efficiency exceeding 9%. Sci Rep. 2012;2:591. 22912919 10.1038/srep00591 PMC3423636

[24] Grätzel MJ. Photoelectrochemical cells. Nature 414, 338–344 (2001). 10.1038/35104607 11713540

[25] Snaith HJ. Perovskites: the emergence of a new era for low-cost, high-efficiency solar cells. J Phys Chem Lett. 2013;4:3623–30.

[26] Xiong L, et al. Review on the application of SnO_2_ in perovskite solar cells. Adv Funct Mater. 2018;28:1802757.

[27] Lu H, Tian W, Gu B, Zhu Y, Li L. TiO_2_ electron transport bilayer for highly efficient planar perovskite solar cell. Small. 2017;13:1701535. 10.1002/smll.201701535 28834132

[28] Hagfeldt A, Boschloo G, Sun L, Kloo L, Pettersson HJC. Dye-sensitized solar cells. Chem Rev. 2010;110:6595–663. 20831177 10.1021/cr900356p

[29] Kim DH, et al. Niobium doping effects on TiO_2_ mesoscopic electron transport layer-based perovskite solar cells. Chemsuschem. 2015;8:2392–8. 25891531 10.1002/cssc.201403478

[30] Xie F, et al. High-efficiency fiber-shaped perovskite solar cells with TiO_2_/SnO_2_ double-electron transport layer materials. J Electron Mater. 2023;52:4626–33.

[31] Guan X, et al. Low-dimensional metal‐halide perovskites as high‐performance materials for memory applications. Small. 2022;18:2203311. 10.1002/smll.202203311 35989093

[32] Zhang H, Liang X, Zhang Y, Chen Y, Park N-G. Unraveling optical and electrical gains of perovskite solar cells with an antireflective and energetic cascade electron transport layer. ACS Appl Mater Interfaces. 2023;15:21152–61. 37073758 10.1021/acsami.3c02233

[33] Wang K, Liang Z, Wang X, Cui XJ. Lead replacement in CH­_3_NH_3_PbI_3_ perovskites. Adv Electron Mater. 2015;1:1500089.

