# Supplementary material for: Numerical optimization of TiO2/SnO2 bilayer electron transport layers for enhanced perovskite solar cell performance
Source: Discov Nano. 2025 Sep 15;20(1):161. doi: 10.1186/s11671-025-04357-w (PMC12436251; doi:10.1186/s11671-025-04357-w)
Supplement: Supplementary file 1 — Supplementary Material 1 [file 11671_2025_4357_MOESM1_ESM.docx]

**Support Information**

**Simulation analysis of electron layer thickness based on tin-based two-electron layer perovskite solar cells**

Haoran Ma^1^, Yajun Xu^1,*^ , Jun Zhao^1^, Jun Wu^1^, Jinjie Zheng^2^, Wei Zhang^3^

^1^School of Electrical and Energy Engineering, Key Laboratory of Optoelectronic Materials, Nantong Institute of Technology, Nantong Jiangsu 226002, PR China

^2^ College of Physics, Nanjing University of Aeronautics and Astronautics, Nanjing 211106, PR China

^3^ Suzhou Huaqinyuan Microelectronics Technology Co. Ltd., Suzhou Jiangsu 215600, PR China

* Corresponding author: yajunxu@ntit.edu.

**Supplementary Figures**

| **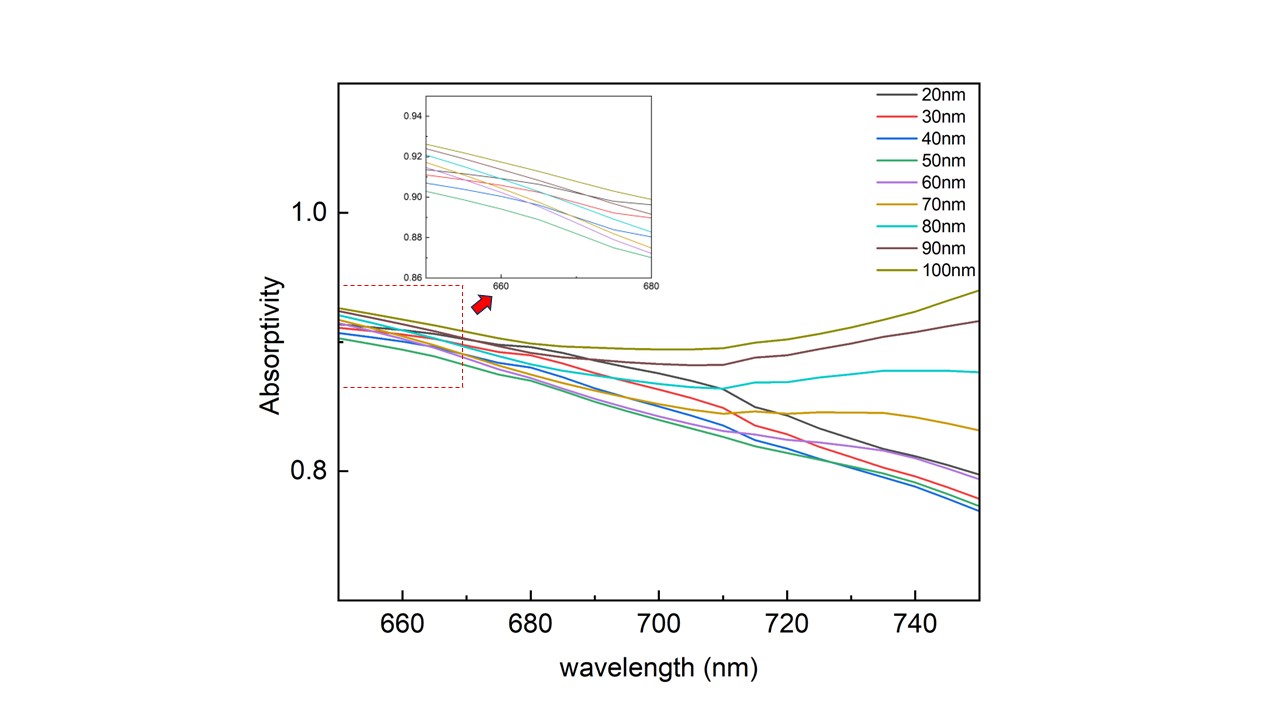** |
| --- |
| **Fig. S1** Absorbance curves of TiO_2_ as a PVK solar cell at PVK layer thicknesses of 20 nm -100 nm. |

| 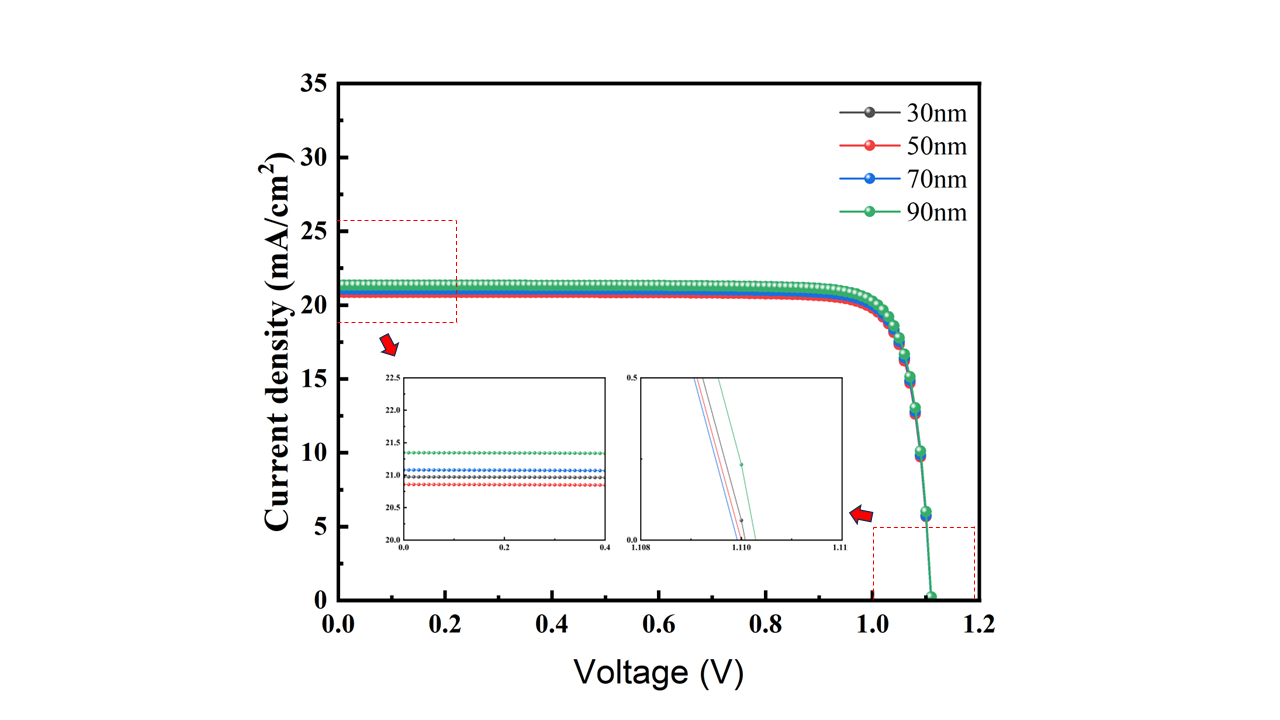 |
| --- |
| **Fig. S2** J-V curves of 30 nm, 50 nm, 70 nm, and 90 nm thickness of TiO_2_ as the thickness of the electronic layer of perovskite cells |


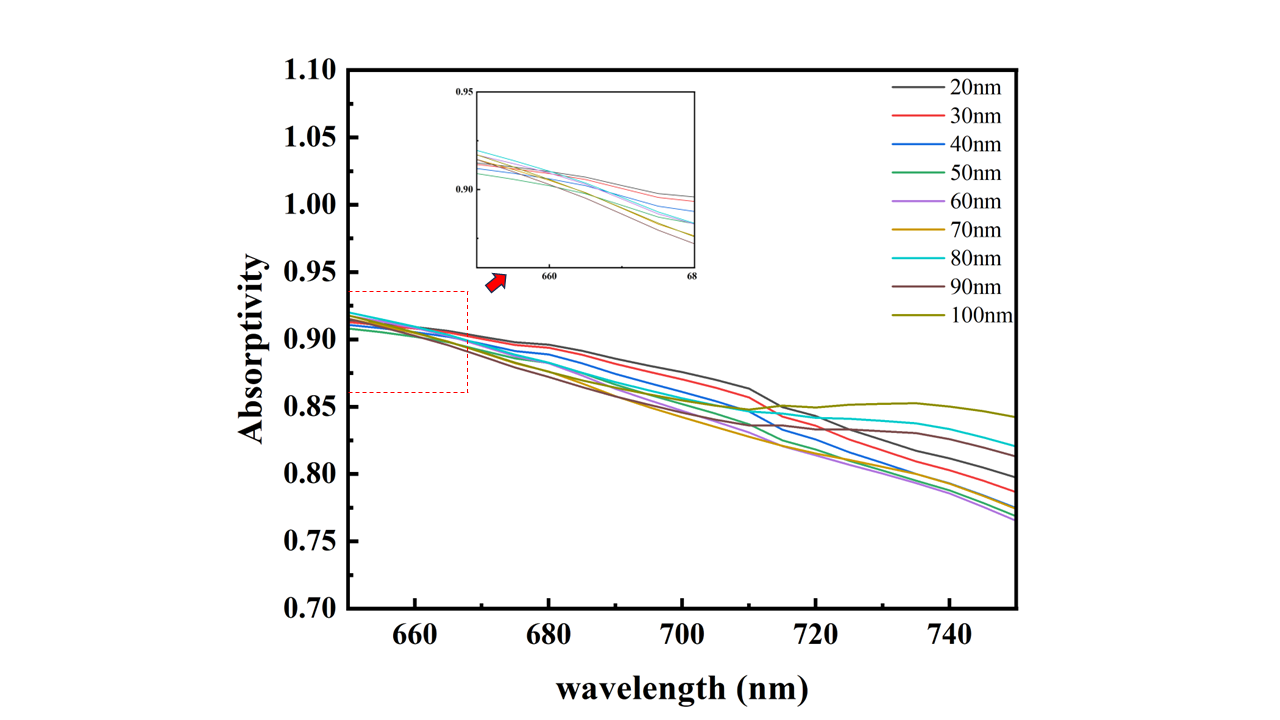


**Fig. S3** Absorbance curves of SnO_2_ as a PVK solar cell at PVK layer thicknesses of 20 nm -100 nm.
